# PLA/Hydroxyapatite scaffolds exhibit in vitro immunological inertness and promote robust osteogenic differentiation of human mesenchymal stem cells without osteogenic stimuli

**DOI:** 10.1038/s41598-022-05207-w

**Published:** 2022-02-11

**Authors:** Marcela P. Bernardo, Bruna C. R. da Silva, Ahmed E. I. Hamouda, Marcelo A. S. de Toledo, Carmen Schalla, Stephan Rütten, Roman Goetzke, Luiz H. C. Mattoso, Martin Zenke, Antonio Sechi

**Affiliations:** 1 National Nanotechnology Laboratory for Agribusiness, Embrapa Instrumentation, Brazilian Agricultural Research Corporation, Rua XV de Novembro, São Carlos, SP 1452 Brazil; 2 Department of Cell Biology, Institute of Biomedical Engineering, RWTH Aachen University, 52074 Aachen, Germany; 3 Department of Hematology, Oncology, Hemostaseology and Stem Cell Transplantation, RWTH Aachen University, 52074 Aachen, Germany; 4 Electron Microscopy Facility, RWTH Aachen University, 52074 Aachen, Germany; 5 Helmholtz Institute for Biomedical Engineering, Stem Cell Biology and Cellular Engineering, RWTH Aachen University, 52074 Aachen, Germany

**Keywords:** Biomaterials - cells, Biomineralization, Mechanical properties, Nanocomposites, Polymer characterization

## Abstract

Bone defects stand out as one of the greatest challenges of reconstructive surgery. Fused deposition modelling (FDM) allows for the printing of 3D scaffolds tailored to the morphology and size of bone damage in a patient-specific and high-precision manner. However, FDM still suffers from the lack of materials capable of efficiently supporting osteogenesis. In this study, we developed 3D-printed porous scaffolds composed of polylactic acid/hydroxyapatite (PLA/HA) composites with high ceramic contents (above 20%, w/w) by FDM. The mechanical properties of the PLA/HA scaffolds were compatible with those of trabecular bone. In vitro degradation tests revealed that HA can neutralize the acidification effect caused by PLA degradation, while simultaneously releasing calcium and phosphate ions. Importantly, 3D-printed PLA/HA did not induce the upregulation of activation markers nor the expression of inflammatory cytokines in dendritic cells thus exhibiting no immune-stimulatory properties in vitro. Evaluations using human mesenchymal stem cells (MSC) showed that pure PLA scaffolds exerted an osteoconductive effect, whereas PLA/HA scaffolds efficiently induced osteogenic differentiation of MSC even in the absence of any classical osteogenic stimuli. Our findings indicate that 3D-printed PLA scaffolds loaded with high concentrations of HA are most suitable for future applications in bone tissue engineering.

## Introduction

Bone is one of the most widely transplanted tissues worldwide, with over 4 million medical interventions per year^1^. The incidence of orthopedic procedures has gained attention due to several aspects, including the increase of life expectancy, trauma, inflammation and tumors^2^. However, the treatment of bone fractures remains a great challenge in the context of surgical procedures^3^. Autografting is currently the standard procedure used for bone repair, however this method has many drawbacks, such as limited tissue availability, pain, donor site morbidity, need for a second surgical treatment, difficulty in producing anatomically shaped grafts, and up to 50% failure rate for specific sites^4^. These disadvantages make the development of engineered implants or scaffolds an urgent requirement. Engineered scaffolds aim to revive bone tissues rather than simply replace them^5^.

Biomaterials have been extensively studied in tissue engineering processes, especially for bone replacement, to replicate the strength, bioactivity, morphology, porosity, and load-bearing ability of living bone tissues, leading to the reconstruction, regeneration and repair of injured bones^6,7^. Ideally, biomaterials should provide a physiological environment for endogenous bone cells, and be resorbed at the same rate at which the new bone tissue is formed^8^. Currently, only a few polymeric biomaterials that are nontoxic, resorbable and approved by the FDA (Food and Drug Administration) are available for the production of scaffolds for clinical use. Among them, polylactic acid (PLA) stands out as a semicrystalline, biobased polyester that displays good biocompatibility and biodegradability^9^. However, the major limitations of PLA that restrict the fabrication of bone scaffolds include its poor mechanical strength and low cell affinity^10,11^. Interestingly, the incorporation of ceramics can overcome the hydrophobicity of PLA, improve its mechanical properties, and stimulate the osteoinduction and osteointegration of the implanted scaffold^12^.

Hydroxyapatite (HA, Ca_5_(OH)(PO_4_)_3_), is categorized as a bioactive, nontoxic, osteoinductive, and osteoconductive ceramic of great importance for bone scaffolds due to its close analogy to the mineral portion of bones and teeth, and its capacity to form direct chemical bonds with living tissues^13^. In this context, the combination of PLA with HA could result in polymer composites with increased level of bioactivity and regeneration potential for bone tissue engineering^14^. In such composites, the PLA phase could provide physical support for cell growth, whereas the HA phase could support cell proliferation and osteoinduction^15^. Furthermore, the incorporation of HA was found to enhance the mechanical properties, fiber diameter and pore size of PLA nanofibers^16^. Kim et al. produced nanocomposite fibers by electrospinning and reported an excellent cell attachment and proliferation^17^. However, electrospun composite fibers are not adequate for producing bone grafts, especially for critical-sized bone defects^18^.

Several technologies have been exploited for fabricating bone grafts based on polymer composite, such as solvent casting, leaching, freeze-drying, thermally induced phase separation and gas foaming^19^. However, these techniques have various limitations, including difficulties in controlling the internal micro-architecture (shape, size and distribution of pores), and the presence of residual cytotoxic solvents^20^. Additive manufacturing techniques, like Fused Deposition Modelling (FDM), are better suited to produce scaffolds individually tailored to fit the morphology of specific tissue defects. The scaffolds can be fabricated by FDM in a controllable way with precise spatial deposition of material components, enabling a customized external shape with predefined internal porosity and interconnectivity to mimic the natural microarchitecture of bones^21^.

Regarding the cellular component of scaffolds for bone repair, mesenchymal stem cells are widely used due to their ability to easily differentiate towards osteoblasts^22–24^. Standard differentiation protocols include the use of osteogenic media additives^25^. However, variations of osteogenic media induce variations in the response of MSC towards osteoblast differentiation, thus calling for media-independent osteogenic stimuli^25^. Furthermore, when designing scaffolds for bone repair, it should be given particular attention to the immunological aspect of their implantation since immune cells, particularly dendritic cells, are known to be influenced, to a large extent, by biomaterials^26,27^.

Taking into consideration the above issues, 3D polymer composites could be designed to exhibit adequate porosity and pore size, degradation rate, mechanical strength, and biological properties tailored for bone tissue engineering^28^ such that the variability introduced by classic soluble osteogenic factors is eliminated. In this context, Soheilmoghaddam and colleagues^29^ have shown that scaffolds composed of poly(lactic-*co*-glycolic acid) (PLGA) and HA scaffolds can support osteogenic differentiation of human MSC under osteogenic-free conditions. Other studies have also developed PLGA-based scaffolds to support human MSC osteogenic differentiation. For instance, PLGA can be combined with PEG^30^, collagen^31^, grafted HA^32,33^, or beta-tricalcium phosphate^34^ to achieve a significant osteoinductive effect. In addition, PLA was combined with gelatin and alginate^35^ or PCL to support osteogenic differentiation of human MSC and C3H10T1/2 cells, without differentiation media^36^. Scaffold pore size is also an important factor with influence at cellular proliferation, ECM production and vascularization. Despite a consensus has not been defined yet, a pore size of 100–500 µm allows the cell penetration and migration, new tissue deposition and nutrient delivery^37,38^.

Even though PLA/HA composites have been widely studied, there are only a few studies regarding the incorporation of high HA loadings in PLA^39–42^. Furthermore, their osteogenic potential and the response of immune cells to PLA/HA composites are still not fully investigated. In this study, we aimed at evaluating for the first time the efficacy of high HA concentrations (between 20 and 25% w/w) on the osteogenic response induced by 3D-printed PLA/HA composites on human mesenchymal stem cells. In addition, we analyzed the influence of the 3D-printed PLA/HA composites on the activation status of dendritic cells, a class of immune cells that play a pivotal role in the immune response. Our findings show that the 3D-printed PLA/HA composites do not induce an active, immune-competent state in dendritic cells. Moreover, all 3D-printed PLA/HA composites induce a robust osteogenic differentiation of human mesenchymal stem cells even in the absence of classical soluble osteogenic stimuli.

## Materials and methods

### Materials

Pellets of poly(lactic acid) (PLA, Ingeo biopolymer 2003D, with 4.3% of d-lactic acid and melt flow index of 6.0 g/10 min (210 °C, 2.16 kg); number average molecular weight (Mn) 100,422 g/mol, average molecular weight (Mw) 180,477 g/mol and polydispersity index, 1.79) were purchased from NatureWorks LLC (Minnesota, EUA). Powder hydroxyapatite was obtained from Sigma-Aldrich (St. Louis, MO, USA), reference number 21223, purity > 90% and d_50_ = 3.73 µm. For other materials, see Supplementary Information S1.

### Preparation of polylactic-based composite filaments

Different PLA/HA weight ratio composites (see Table S1) were obtained using the solution casting method, as described in Bernardo et al.^14^. Filaments suitable for 3D printing were obtained by melt extrusion, using a twin-screw extruder (Baker and Perkins, Process Equipment and Systems) divided into 5 heating zones running at 180 °C. The screw diameter was 19 mm, and the L/D of the extruder was constant and equal to 25 mm. The molten filament was immediately cooled in a water bath at the circular die outlet (2 mm in diameter). To obtain a filament with diameter compatible for the 3D printer (D = 1.75 mm), the stretching speed of the molten filament was adjusted for each composition. Printing was performed using a Cliever Cl1 3D printer (Brazil) using the following printing parameters: nozzle size, 0.3 mm; layer height, 0.1 mm; distance between layers, 0.25 mm. The nozzle and table temperatures were set at 180 °C and 50 °C, respectively.

### Characterizations

The methods for characterization of filaments and 3D-printed scaffold are reported in Supplementary Information S1.

### Mechanical tests

Tensile tests were carried out following the ASTM D638-14 standard (Fig. S1A) on the universal testing machine EMIC DL3000 (EMIC Equipamento & Ensaio Ltda, PR, Brazil) equipped with a 500 kgf (4903.32 N) load cell. The true tensile strength (σ_T_), true elongation break (ε_B_) and Young's modulus (E_T_) of the scaffold samples were determined from stress (σ)–strain (ε) curves. σ_T_ was calculated as σ_T_ = σ λ, where σ is the engineering tensile strength and λ denotes the extensional ratio defined as λ = L/L_0_, where L and L_0_ represents the final and initial specimen lengths, respectively. ε_B_ was obtained as ε_B_ = ln λ, while the Young's modulus (E) was determined through linear regression of the σ–ε curves in the limit σ = ε = 0 ([dσ/dε]ε = 0). Average scaffold thickness was determined measuring five random positions in each specimen using a digital micrometer (Mitutoyo Manufacturing, Japan). Compression tests were performed following the ASTM D695-10 standard (Fig. S1B) on the universal testing machine EMIC DL3000 equipped with 3000 kgf (29,419.25 N), with head velocity of 1.0 mm min^−1^. Young's modulus (Ec) and yield strength (σ_c_) under compression were calculated similarly to the properties calculated from the tensile tests. Each mechanical test (tensile and compression) was performed with at least 10 replicates.

### Tests of degradation

The degradation behavior of 3D-printed polymer composites was characterized through in vitro degradation tests, as described in the Supplementary Information S1.

### Generation and culture of dendritic cells

Murine dendritic cells were cultivated from bone marrow progenitor cells isolated from mouse hind legs as described previously^26^ (Supplementary Information S1). All animal experimental protocols were approved by local authorities at RWTH Aachen University in compliance with the German animal protection law and EU guidelines (2010/63/EU) for animal protection, as well as all methods were carried out in accordance with the relevant guidelines and regulations. Anesthesia and euthanasia were performed in the context of paragraph 4, section 3 of the German animal welfare act (killing of animals for scientific purposes). Additionally, the study was carried out in compliance with the ARRIVE guidelines.

### Culture of human mesenchymal stem cells (MSC)

MSC were isolated from the bone marrow of two donors (28 and 68 years of age) after orthopedic femoral head replacement surgery. All samples were taken after written informed consent. The study was approved by local ethics committees of RWTH Aachen University (RWTH Aachen; EK128/09). All experimental protocols were performed in accordance with relevant guidelines and regulations of the RWTH Aachen University. MSC were isolated and cultivated as described before^43^ (Supplementary Information S1).

### In vitro biocompatibility

Before all biological assessments, the 3D-printed scaffolds (7 mm × 7 mm × 1 mm, height x length x thickness, with pore size of 300 µm; see Fig. S2) were sterilized under UVC radiation (λ = 254 nm) in a laminar flow cabinet for 30 min and incubated with cell culture medium for at least 24 h. The cytotoxicity of the 3D printed samples (PLA, 80:20S and 75:25S) was quantified using the (3-[4,5-dimethyl- 2-thiazol]-2,5-diphenyl-2H-tetrazolium bromide) (MTT) assay, as described in the Supplementary Information S1.

### FACS analysis

Briefly, immature dendritic cells (1 × 10^5^ cells/scaffold) were incubated on scaffolds for 24 h or 48 h, while MSC (1.5 × 10^5^ cells/scaffold) were incubated for 14 or 21 days in a 48-well plate at 37 °C, 5% CO_2_. Cells seeded on empty wells (without scaffolds) served as the controls. Afterwards, cells were gently removed from the scaffold surface, collected and washed with FACS buffer (PBS supplemented with 0.5% FCS and 2 mM EDTA). The cells were resuspended in 50 µL FACS buffer and incubated with the selected antibodies for 30 min at 4 °C. The cells were then washed with 1 mL of FACS buffer, re-suspended in 200 µL FACS buffer and kept on ice. The following surface proteins were analyzed: for dendritic cells MHC-II, CD40, CD80 and CD86, and for MSC CD10, CD105 and CD90. In both cases, surface marker expression was analyzed by flow cytometry using a FACS Canto II (BD Bioscience, Germany). Data were collected from 50,000 viable cells and analyzed using FlowJo software (FlowJo, LLC, USA). All experiments were repeated in duplicates using three replicates for each experiment.

### RNA isolation and RT-PCR

RNA isolation and cDNA synthesis were performed as previously described^26^. For dendritic cells, the expression of the cytokines IL-1β, IL-6, IL-10, IL- 12p40, and RANTES was analyzed after 24 or 48 h of incubation with the scaffolds. Immature dendritic cells were treated with 1 µg/mL LPS and served as the positive controls. For MSC, the expression of BMP-2, runt related gene-2 (RUNX-2), osteocalcin (OCN), and type I collagen (COL1A1) was assessed after 7, 14 or 21 days of culture with the scaffolds. In both cases, gene expression was determined by RT-qPCR (Thermocycler, Eppendorf, Hamburg, Germany) using the primers listed in Table S2. The relative quantification of each targeted gene was normalized to GAPDH levels and calculated via the 2^–ΔΔCt^ method. All experiments were performed twice including three replicates for each experiment.

### MSC differentiation on scaffolds

To determine MSC differentiation, cells were seed at the density of 1 × 10^5^/scaffold (at passage 5) on sterile scaffolds. The medium was replaced 24 h after seeding for determining the MSC differentiation under osteogenic conditions. The medium was refreshed every second day over 21 days of analysis. For ALP (alkaline phosphatase) quantification, cells were lysed with 1% Triton X-100 solution. Immediately after lysis, the protein content was determined using the Bradford Protein Assay, as reported elsewhere^44^. The ALP concentration was determined according to a previously reported procedure^45^ (Supplementary Information S1).

Alizarin Red staining was used to evaluate the MSC mineralization at day 21 of the osteogenic differentiation. Cells were washed three times with PBS, fixed with 4% paraformaldehyde (PFA) for 15 min, stained with alizarin red solution (0.5%, Sigma-Aldrich, MO, USA) for 20 min at 37 °C, and then washed several times with deionized water to remove the excess of stain. The images were acquired with an Evos Microscope (Thermo Fisher, Germany). For the quantification of Alizarin Red, the stain was removed using 10% of acetic acid solution for 30 min. Afterwards, the solution thus obtained was transferred to an Eppendorf tube and heated at 85 °C for 10 min, followed by centrifugation at 12.500 rpm for 15 min. Then, 100 µl of the supernatant was used for absorbance measurements at 405 nm using a microplate reader (Molecular Devices SpectraMax M2e). For quantification, background staining from scaffolds without cells was subtracted from the values obtained from the scaffolds with cells. All experiments were repeated in triplicate.

### Confocal and scanning electron microscopy

For confocal microscopy, cells were fixed with 4% PFA for 30 min at 25 °C and then permeabilizated with 0.1% Triton X-100 for 1 min. The samples were stained with Alexa Fluor 647-phalloidin (Sigma- Aldrich, St. Louis, MO) to visualize the actin cytoskeleton. All samples were mounted on glass slides using ProLong (Molecular Probes Life technologies). Images were acquired using a LSM700 confocal microscope (Zeiss, Germany) equipped with a 63X oil immersion objective and 633 nm laser line. Confocal images were processed using ImageJ. The SEM imaging was carried out as reported elsewhere^46^.

### Statistical analysis

Data were subjected to analysis of variance and Duncan multiple or Games-Howell comparation test using the software R, version 3.6.0 (https://www.R-project.org/). The Games-Howell method was adopted following the variance heterogeneity of samples regarding the mechanical property deformation at break. The Bartlett test was used to verify the variance homogeneity condition. For the biological studies, the statistical analyses were done with Prism 8 (GraphPad, Software Inc., USA) using the one-way ANOVA in combination with the Tukey method with a statistically significant difference set at p < 0.05.

## Results and discussion

### Characterization of PLA-HA filaments and 3D-printed scaffolds

Thermal analysis is essential to characterize the behavior of the PLA-HA polymer composites. DSC showed that for both, first and second heating scans, the glass transition temperature (T_g_) of the PLA-HA composites decreased in comparison with that of neat PLA (Table 1; for the DSC curves, see Fig. S3). In this context, the increase of HA amount may increase the interfacial area with PLA, thus affecting the mobility of the polymer chains and leading to the decrease of the T_g_ in the PLA-HA composites^47^. The slight difference observed between the T_g_ of 80:20S and 75:25S composite could be explained by the different distribution of HA particles. In 75:25S composites, the HA particles may be more uniformly distributed leading to stronger interfacial interactions between HA particles and polymer chains, thus increasing the chain organization and, therefore, the glass transition temperature, in comparison to the 80:20S^48^. In addition, the formation of homogenous crystals likely caused the reduction in the melting temperature (T_m_) (*i.e.,* less energy is required to melt the PLA chains) of the PLA/HA composites (Table 1). It is also important to note that the crystallinity degree was higher for the PLA/HA polymer composites than for neat PLA (Table 1) suggesting that HA particles may have acted as a nucleating agent, thus supporting the increase in the crystallinity, which could result in the improvement of the mechanical properties of composites^49,50^. This aspect is critical for bone replacement applications since the mismatch of the mechanical properties between the bone tissue and the scaffolds could lead to implant failure.

**Table 1:** DSC data of neat PLA and PLA-HA polymer composite filaments.

Samples	First heating scan			Second heating scan					
	T_g_ (°C)	T_m_ (°C)	T_cc_ (°C)	T_g_ (°C)	T_m_ (°C)	T_cc_ (°C)	ΔH_m_ (J/g)	ΔH_c_ (J/g)	*X* (%)
PLA	59.7	150.4	116.8	58.0	151.5	121.7	13.7	10.0	3.9
80:20 S	52.1	145.1	110.6	55.0	148.9	125.0	15.1	09.5	7.4
75:25 S	56.8	146.0	109.4	55.7	150.8	128.1	13.1	08.4	6.7

*T*_*g*_ glass transition temperature, *T*_*cc*_ cold crystallization temperature, *T*_*m*_ melting temperature, *ΔH*_*m*_ enthalpy of melting, *ΔH*_*c*_ cold enthalpy, *X* crystallinity degree.

TGA analysis of the 3D-printed scaffolds enabled the precise determination of the HA percentage in the composites, and the influence of HA content on the degradation temperature of neat PLA and the thermal stability of the printed samples (Fig. S4). The temperature of the maximal degradation rate of the 3D-printed polymer composites was 25–30 °C higher than that for neat PLA. This may be related to the carrier effect of HA toward the polymer decomposition ablation products^49^. It is important to note that the degradation temperature for all samples was significantly higher than the printing temperature. Thus, the polymer composites should not undergo any degradation during the 3D-printing process. The inorganic content of the 3D-printed samples corresponded to the expected HA contents indicating that the extrusion and 3D-printing process had no effect on the amount of HA in the polymer composites.

### Mechanical properties of 3D-printed composites scaffolds

Tensile and compression tests were carried out to assess the mechanical strength and elastic modulus of the 3D-printed scaffolds (Table 2). Both, tensile and compression elastic moduli, significantly increased with the presence of HA. As expected, in 3D-printed composites the tensile elongation at break decreased as the elastic modulus increased in comparison to neat PLA. The PLA/HA composites were found to have a slight difference in tensile strength compared to the neat polymer. Moreover, the compressive yield strength of the composites also increased indicating that the PLA/HA composites were more mechanically resistant than neat PLA. These results may be related to the increase of the crystallinity of PLA/HA composites^51^ (Table 1) and to the good dispersion of HA particles at the composites^52^.

**Table 2:** Mechanical properties of 3D-printed scaffolds.

Samples	Tensile properties			Compressive properties	
	E_T_ (MPa)	σ_T_ (MPa)	ε_B_ (%)	E_c_ (MPa)	σ_c_ (MPa)
PLA	96.2 (3.2)^b^	25.52 (1.1)^a^	0.07 (0.003)^a^	108.1 (1.6)^c^	42.06 (0.6)^b^
80:20S	117.7 (7.3)^a^	20.35 (3.1)^bc^	0.06 (0.006)^b^	160.2 (6.7)^a^	61.67 (3.4)^a^
75:25 S	121.4 (7.3)^a^	20.62 (1.9)^c^	0.05 (0.004)^b^	125.0 (6.2)^ab^	49.40 (0.9)^a^

*E*_*T*_ tensile elastic modulus, *σ*_*T*_ tensile strength, *ε*_*B*_ elongation at break, *E*_*c*_ compression elastic modulus, *σ*_*c*_ compressive yield strength.

Mean value (standard error). Means in the same column bearing the same letter are not significantly different (p > 0.05).

Ideal bone substitutes should exhibit mechanical properties similar to those of the bone tissue that they are intended to substitute^53^. It is worth noting that the measured compression elastic modulus of the PLA/HA composites were comparable to the values reported for trabecular bone (129.07 ± 49.48 MPa)^54^. We envisage that further tuning of the properties of PLA/HA composite could lead to compression elastic moduli matching those of the more compact cortical bone. Hence, this achievement overcome one of the main limiting factors for real applications of 3D-printed composite scaffolds: the mismatch of elastic modulus with the surrounding bone, which can lead to implant failure.

Porosity is a key factor for an efficient scaffold/tissue interaction, because cell infiltration, nutrient diffusion and proper vascularization of the forming tissue are facilitated in porous scaffolds^19^. Table S3 shows the porosity values of the 3D-printed polymer composite scaffolds (Fig. S1D). Generally, it is expected that an increase of the porosity leads to a decrease of the mechanical properties, due to the reduced number of support points and the overall contact area between the layers of the 3D-printed scaffold, thus increasing the scaffold brittleness^37^. The presence of HA increased the porosity of the 3D-printed scaffolds by a factor of 3, whereas the tensile strength and elongation at break were only slightly reduced. However, the other mechanical parameters were not decreased due to increased porosity. In contrast with previous studies^54–56^, we observed an increase of most of the mechanical properties of the 3D composites and, at the same time, an increase of their porosity. Nevertheless, after implantation, a higher porosity could improve the mechanical interlocking between the scaffold and the surrounding biological tissue, providing greater mechanical stability of the of scaffold/bone tissue interface^57^. Therefore, scaffolds with the highest porosity at the expense of a slightly reduced mechanical properties are to be preferred as substitute of injured bone tissues, as in the case of the 3D-printed scaffold with 20% HA.

### In vitro degradation

**Figure 1:**
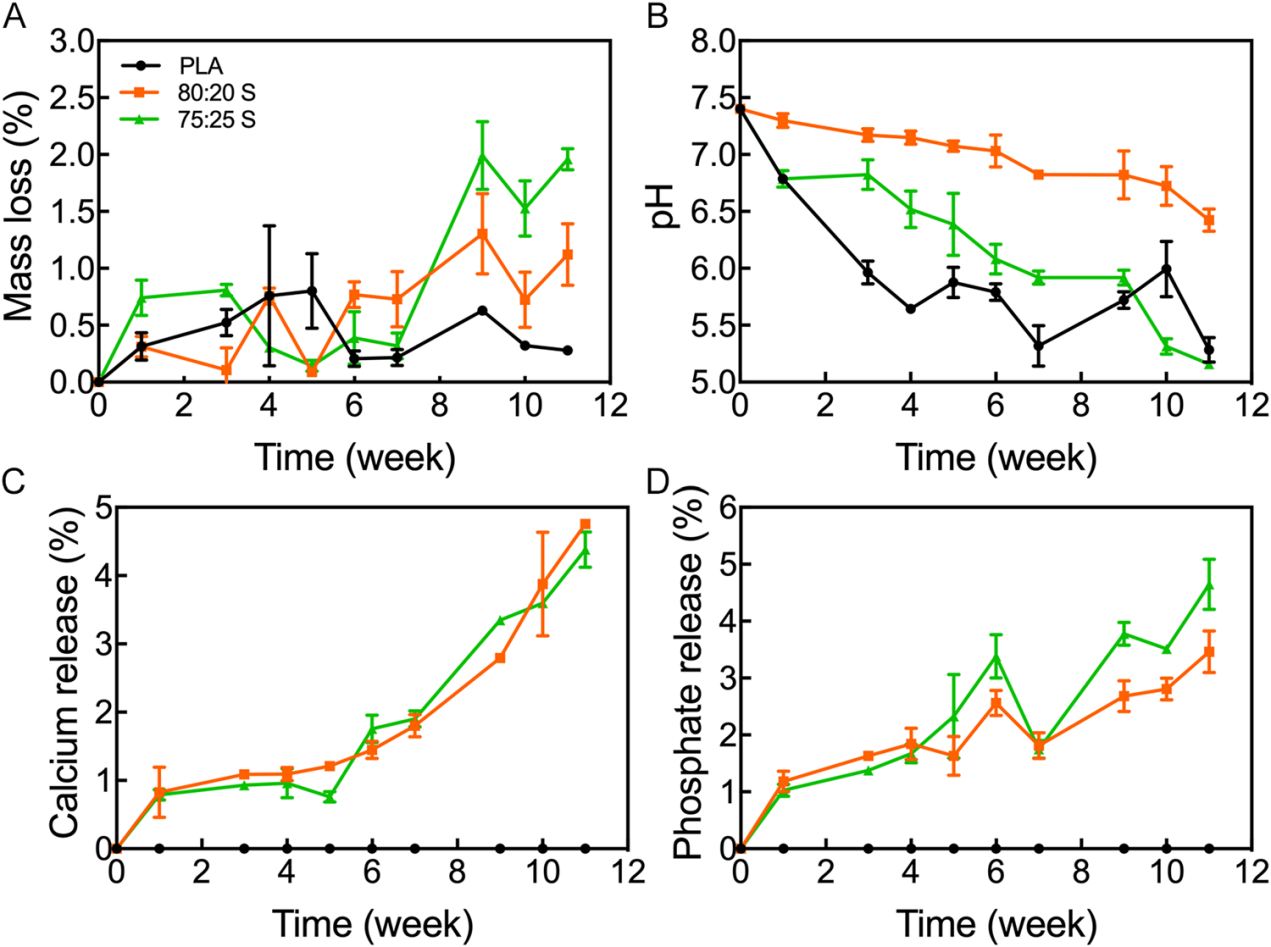
In vitro analysis of (**A**) mass loss, (**B**) pH change, (**C**) calcium and (**D**) phosphate release for 3D-printed scaffolds. Error bars indicate one standard deviation above and below the means.

An ideal scaffold should be characterized by a balanced resorption rate such that space for the newly forming bone tissue is created in a timely manner^58^. Therefore, we investigated the evolution of mass loss, pH change, calcium and phosphate release from the 3D scaffolds over a period of 11-week (Fig. 1).

All scaffolds underwent an increasing mass loss over time, but each scaffold exhibited a specific behavior. For the PLA/HA composite scaffolds, the degradation rate increased with increasing the HA content. Nevertheless, the PLA/HA scaffolds exhibited higher degradation rates rather than neat PLA (Fig. 1A). The incorporation of HA improved the hydrophilicity of the scaffold, resulting in an accelerated degradation process (For contact angle images, Fig. S5). Also, the composite scaffolds exhibited increased porosity, which favors water absorption and therefore increases the degradation rate. As the PLA/HA composite scaffolds, showed similar porosity, both samples ascribes a superior degradation rate in comparison to neat PLA, but no changes were noticed among the samples^28,59^.

The PLA hydrolysis generates acidic products, mainly lactic acid, that decreases the solution pH, as observed in Fig. 1B. The decrease in pH could accelerate the degradation in an autocatalytic way, as observed for the neat PLA scaffold. The PLA/HA scaffolds also presented a decrease in pH over time, but the decreasing rate was slower due to the HA neutralization effect. As the mass loss percentage was low for the 80:20S, the acidification effect was easily neutralized by the HA particles. The increased HA content in the 75:25S led to high mass loss percentage, followed by the increase of PLA degradation, thus generating greater acidification. Nevertheless, the HA has also a neutralizing effect at the solution pH. Low pH values (below 7.4) could negatively influence the bone tissue development, whose cells are pH sensible^42^. Thus, 20% HA content are ideal for the 3D-printed PLA/HA scaffolds, due to the intermediate degradation rates that lead to adequate HA solubilization in the medium to neutralize the excess of lactic acid. 20% HA contents also provide the required environment and elements for bone tissue regeneration (Fig. 1C,D), in addition to the adequate mechanical properties and porosity for the scaffold.

### 3D-printed scaffolds do not activate dendritic cells

Dendritic cells (DC) are the most potent antigen-presenting cells that activate T-cells and bridge innate and adaptive immunity. In light of future applications of the PLA/HA scaffolds as bone replacements, it is important to test if scaffolds trigger an immune response. To this end, we incubated DC with the scaffolds for 24 or 48 h at 37 °C. The choice of these incubation times was dictated by the limited life span of DC^60^. Untreated cells (not exposed to scaffolds) were used as the control. We found that the viability of DC was not affected by incubation with all 3D-printed scaffolds, indicating an excellent biocompatibility of the scaffolds towards DC (Fig. S6).

**Figure 2:**
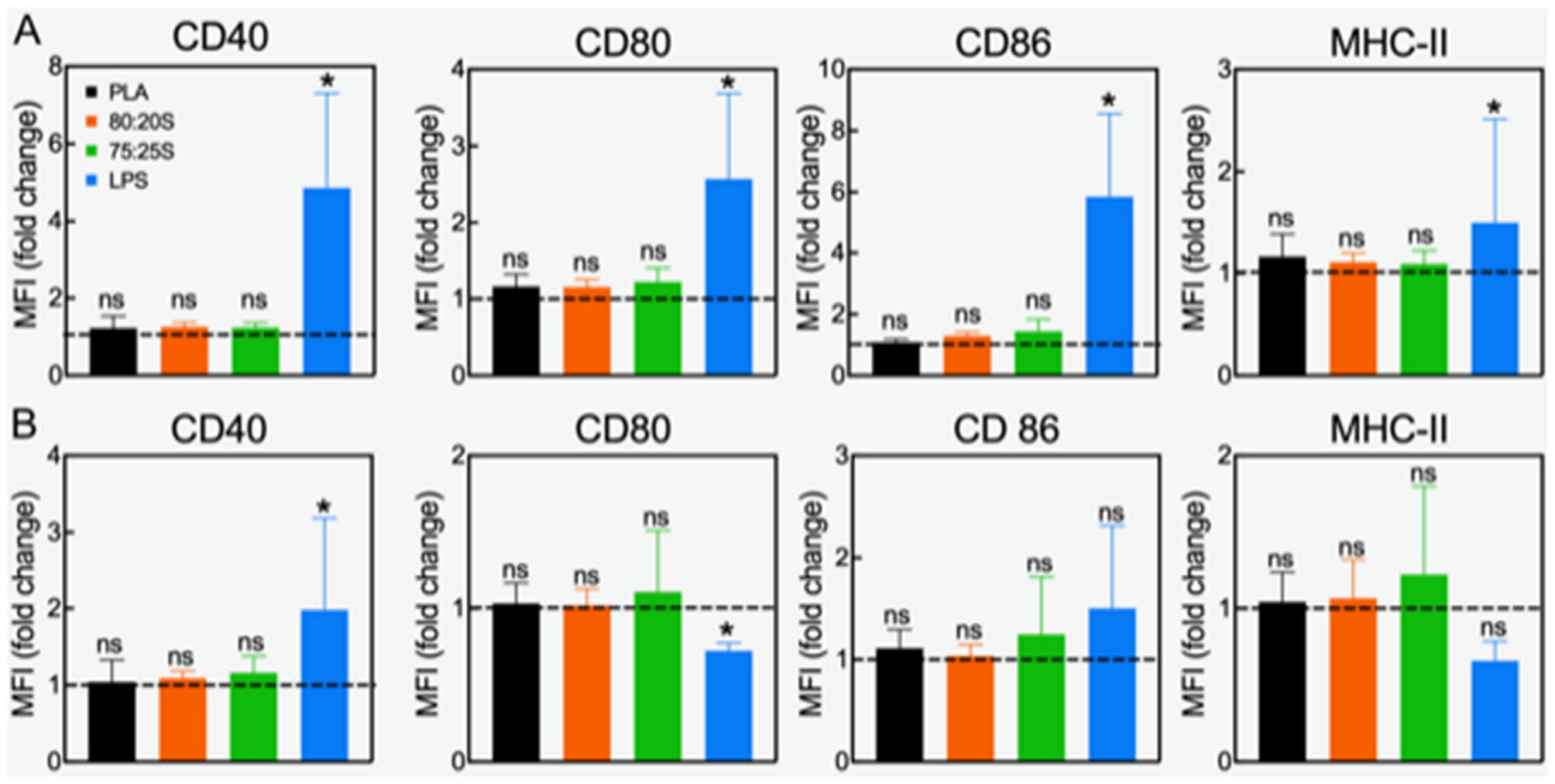
Surface markers expression of DC exposed to 3D-printed scaffolds for 24 (**A**) or 48 h (**B**). Results of two independent experiments using three-well replicates for each repetition are shown. Lines set to 1 represent the control (immature not activated DC). *Ns* no statistical difference; *p < 0.05. Please note that the statistical analysis refers to the comparison with the control.

Several biomaterials can induce immune reactions, despite their frequent use in clinical applications^46^. From the perspective of using the 3D-printed scaffolds as bone substitutes, we aimed at evaluating the functional status of DC based on two parameters, namely, the surface markers and cytokine expression levels. Immature DC express very low levels of surface activation markers, such as CD 40, CD 80, CD 86, and MHC class II, and do not have notable expression of cytokines, such as IL-6, IL-10, IL-12p40, RANTES, and IL-1β^61^. As shown in Fig. 2, the expression levels of the selected surface markers showed no increase after 24 or 48 h of DC incubation with all 3D-printed scaffolds, in comparison with untreated DC, indicating that PLA and PLA/HA scaffolds do not activate DC. Treatment with LPS (a bacterial cell wall component) was used as reference for DC activation and showed a robust increased of all surface markers.

**Figure 3:**
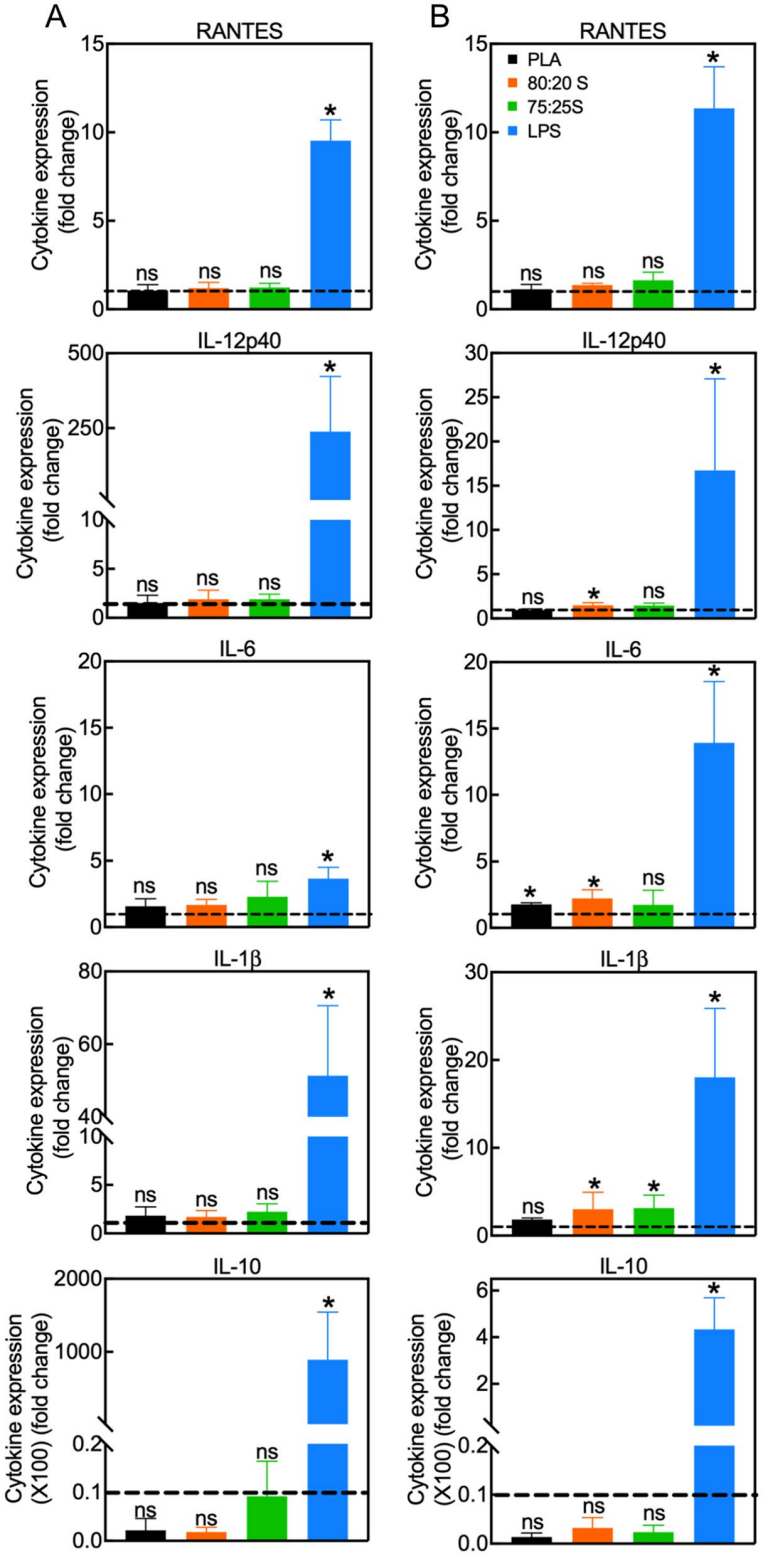
Quantification of cytokine expression in DC incubated with 3D scaffolds for 24 (**A**) or 48 h (**B**). Data show the mean ± SD of two independent RT-PCR measurements. All values were normalized to the expression levels of GAPDH. Gene expression in control DC (cultured on tissue culture dishes) was set to one (dashed lines). LPS-treated DC served as the positive controls. *p < 0.05, *ns* non-significant. Please note that the statistical analysis refers to the comparison with the control.

Next, we investigated the impact of 3D-printed scaffolds on DC activation by analyzing gene expression of cytokines by RT-qPCR analysis (Fig. 3). In these studies, LPS stimulation was also used as control for DC activation. As for the analysis of surface markers above, the expression levels of the cytokines in DC incubated with the scaffolds were the same as untreated cells (control). DC can be expected to be activated by exogenous biomaterials due to their essential function to sense foreign materials. In fact, some authors have reported that PLA can activate DC due to the PLA surface hydrophobicity^62^. Similarly, DC cultured in a medium with high calcium content showed enhanced DC expression of MHC-II, CD 86 and IL-1β^63^. On the other hand, it has been also demonstrated that the biomaterials-DC interactions are influenced by physical and chemical properties of biomaterials, such as spatial structure, topography, and composition^64^. In this work, we show that the 3D-printed scaffolds composed of neat PLA or PLA/HA, at different PLA/HA weight ratio, do not lead to upregulation of the activation surface markers or inflammatory cytokines in DC. Thus, the unaffected DC state following interaction with scaffolds supports the application of such 3D scaffold for bone tissue replacement applications.

### 3D-printed scaffolds are well tolerated by MSC and promote MSC adhesion

The biocompatibility of 3D-printed PLA and PLA/HA scaffolds toward human MSC was assessed by incubating MSC with scaffolds under normal MSC growth condition for up to 21 days according to the ISO 10993-5:2009 protocol (Fig. S7). In line with the excellent biocompatibility of the scaffolds toward DC, MSC viability was also unaffected by PLA and PLA/HA scaffolds. This observation confirms that all 3D-printed scaffolds are not cytotoxic, which is a fundamental property for tissue engineering applications^8^.

Osteogenic differentiation of MSC involves three main stages: (1) proliferation; (2) extracellular matrix (ECM) synthesis and maturation; (3) ECM mineralization^65^. Each stage is characterized by specific markers, which allow to follow the time course of the osteogenic differentiation.

**Figure 4:**
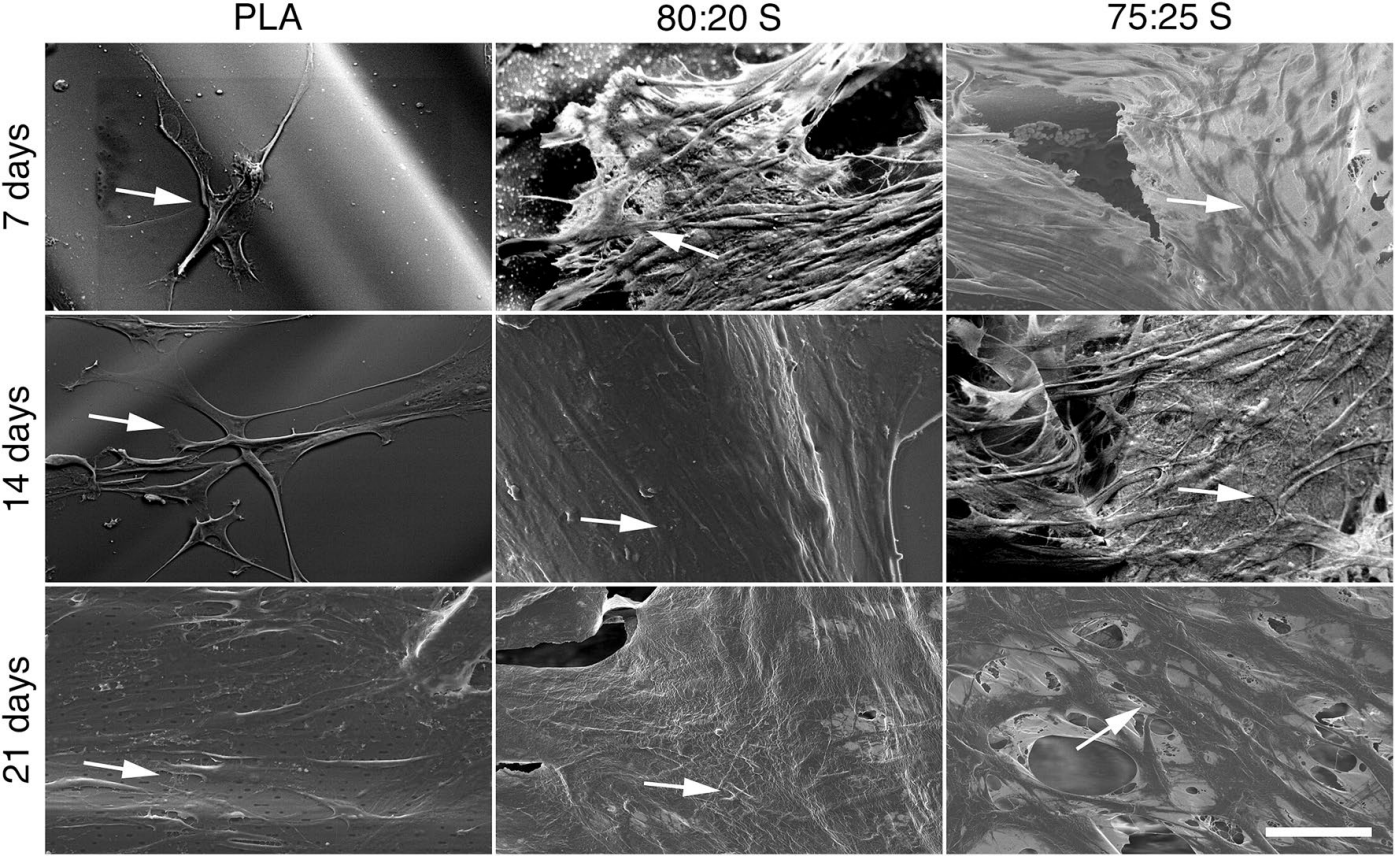
SEM images showing the MSC adhesion on 3D-printed scaffolds after 7, 14 and 21 days of incubation. The arrows point to adherent MSC. Scale bar (for all panels): 50 μm.

**Figure 5:**
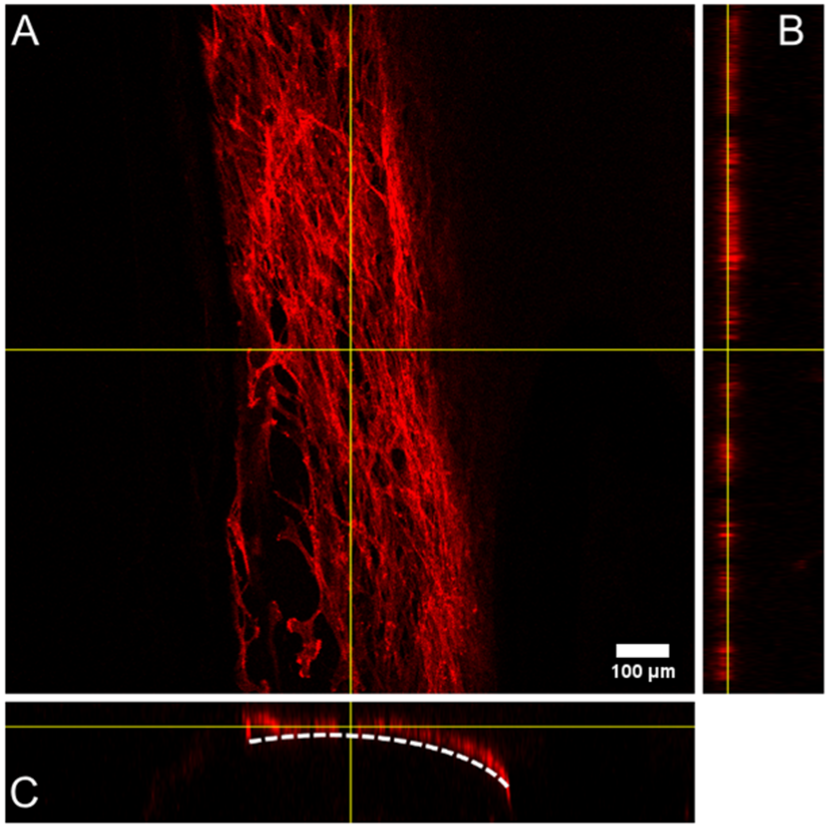
Representative confocal microscopy images of MSC growing on PLA 3D-printed scaffold surface after 7 days of cultivation for (**A**) X–Y plane; (**B**) Y–Z plane; (**C**) X–Z plane. Cells were visualized with Alexa 647-conjugated phalloidin. The dashed line represents the surface of the scaffold. Direct imaging of PLA/HA 3D-printed scaffold was not possible due to their high intrinsic fluorescence.

Cell adhesion is a critical factor for cell proliferation on the scaffold surface^66^. SEM analysis clearly show that MSC efficiently adhere to the scaffold surface, and gradually change their cell morphology, from a spindle-like shape with round nuclei at day 7 (Fig. 4, indicated by an arrow) to an elongated shape with an increased number of cellular extensions after 14 and 21 days of culture. Accordingly, confocal microscopy imaging of MSC stained with fluorescent phalloidin to visualize the actin cytoskeleton showed the tight juxtaposition of adjacent cells that formed more than one cell layer at some locations along the scaffold surface (Fig. 5; for a three-dimensional view, see Supplementary Video S1; for actin cytoskeleton organization at days 14 and 21, see Fig. S8).

### 3D-printed scaffolds promote osteogenic differentiation of MSC in the absence of osteogenic stimuli

The second differentiation stage of MSC involves ECM synthesis, when cells exhibit the highest alkaline phosphatase (ALP) activity. ALP contributes to the initiation of osteoblast mineralization, at the early stages, catalyzing the hydrolysis of organic phosphate to inorganic phosphate, which is a key factor for mineral deposition^66,67^. The effects of the 3D-printed scaffolds on ALP activity were evaluated after incubation of MSC with the scaffolds for 7, 14 or 21 days under growth or osteogenic differentiation conditions (Fig. S9). MSC incubated with the scaffolds under growth conditions showed ALP activity at day 14, increasing by twofold (compared to control), and then ALP activity level decreased at a later time point (21 days), in accordance with previous studies on in vitro development of osteoblastic phenotype^23,68^.

Our observation clearly suggest that 3D scaffolds exert an osteogenic stimulus on MSC even in the absence of classical soluble osteogenic factors. In contrast, in the presence of osteogenic medium, ALP activity peaked at an earlier time point (day 7) and then decreased at later stages. Therefore, even under osteogenic culture conditions, 3D PLA/HA scaffolds promoted higher ALP activity (approximately twofold increase compared to control) than neat PLA scaffold. Several studies described the positive influence of HA or calcium phosphates on ALP activity^68–72^. Thus, under both culture conditions, PLA/HA scaffolds can promote the commitment of MSC toward osteogenic differentiation.

**Figure 6:**
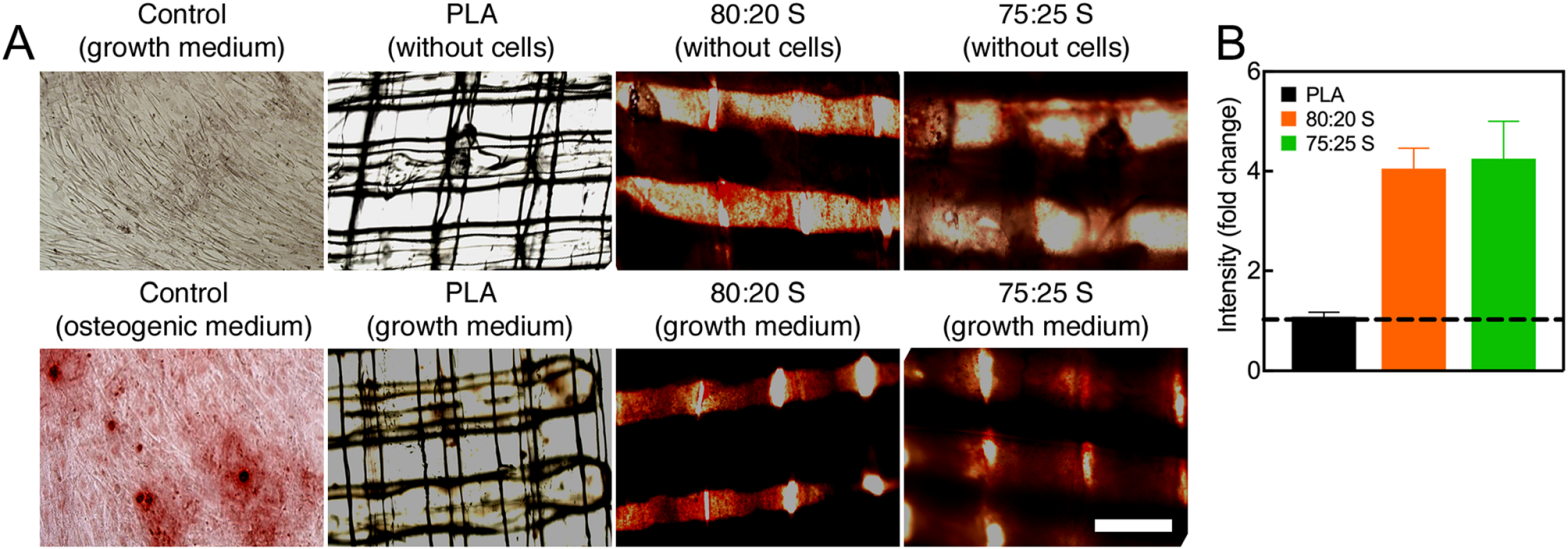
(**A**) Evaluation of osteogenic differentiation of MSC on 3D-printed PLA/HA scaffolds using Alizarin Red staining. MSC were cultured on scaffolds for 21 days under growth conditions. Note the more intense Alizarin Red staining in the lower panels. (**B**) Quantification of Alizarin Red staining after elution and absorbance measurement at 405 nm. The line (set at 1) represents Alizarin Red staining level of control without scaffold. Error bars show one standard deviation above the mean. Scale bar (for all panels): 500 μm.

ECM mineralization is considered a marker for the final stage of the osteogenic differentiation. During this process, calcium deposits are formed and reach their maximum after 2–3 weeks of culture^73,74^. Following incubation of MSC on neat PLA and PLA/HA scaffolds in growth medium for 21 days, Alizarin red staining showed a higher level of mineralization in MSC seeded on PLA/HA as compared to cells seeded on neat PLA scaffolds (Fig. 6 and Fig. S10). Other studies also report that neat polymers, such as PCL^72^ and PLGA^8^, exhibited low or similar mineralization levels as MSC seeded on plastic (control). The strong effect of the PLA/HA scaffolds on the ECM mineralization could be explained by the dissolution of HA in calcium and phosphate ions, which play essential roles in bone metabolism. Calcium is known to support cell adhesion, proliferation, differentiation, and ECM mineralization, while phosphate regulates cell proliferation and stimulates the expression of key proteins involved in the ECM mineralization process^75^.

### 3D scaffolds promote the expression of osteogenic genes in MSC

**Figure 7:**
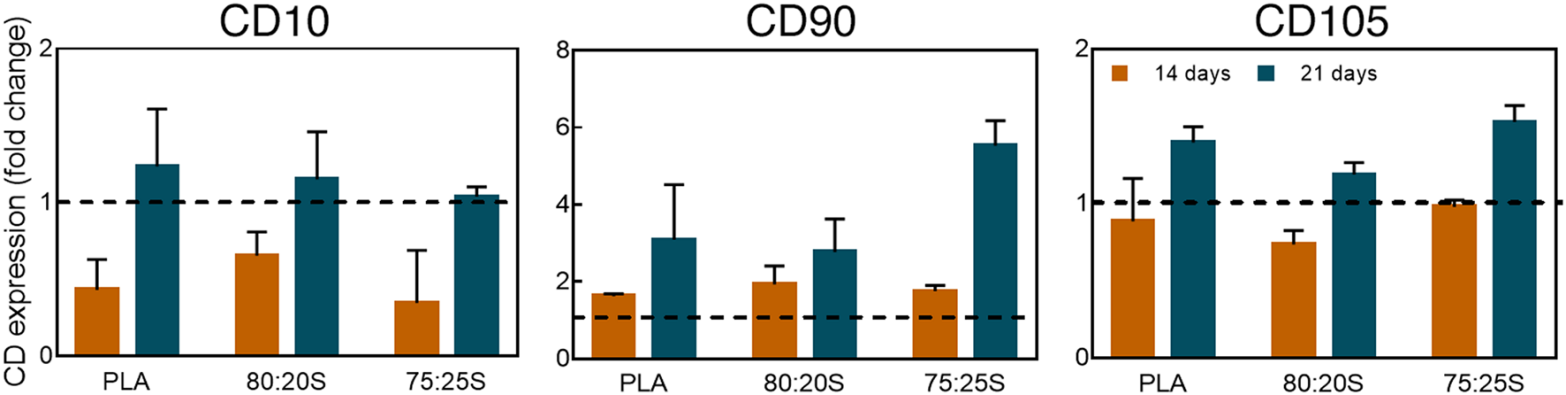
Flow cytometry analysis of CD10, CD105 and CD90 expression in MSC cultured on 3D-printed scaffold for 14 or 21 days under growth conditions. Dashed lines indicate surface marker expression (set to 1) in control cells cultured on tissue culture dishes. Error bars show one standard deviation above the mean.

Next, flow cytometry analysis was performed to evaluate the expression of MSC surface markers during the osteogenic differentiation. Although osteoblasts do not express specific cluster of differentiation (CD) proteins, CD10 surface marker is commonly reported as up-regulated in osteoblasts^76^. In MSC cultured for 14 or 21 days on the 3D-printed scaffolds under growth conditions the expression of CD10 increased over time, thus showing MSC differentiation towards osteoblasts (Fig. 7). A similar finding on CD10 expression was observed under osteogenic condition (Fig. S11). Another surface marker evaluated in this study was CD90, which is frequently considered as a reference marker for MSC. Interestingly, CD90 has also been described as a possible marker of osteoblastic differentiation expressed during the proliferation phase^77,78^. Under the growth conditions (MSC on scaffolds in growth medium), CD90 expression clearly increased peaking after 21 days (Fig. 7). Thus, the concomitant increase in CD 10 and CD90 expression clearly indicates that MSC are committed toward osteogenic differentiation. Finally, the expression of the antigen CD105, a typical marker of MSC^79,80^, was also analyzed. CD105 is a modulator of cellular response upon stimulation with the growth factor TGF-β^81^. Leyva-Leyva et al.^82^ showed that MSC with osteogenic differentiation potential express CD105 while simultaneously exhibiting effective mineral deposition. Herein, a moderate increase of CD105 expression over time was observed. Thus, FACS analysis of surface markers corroborated the above results, indicating a unique effect of the PLA/HA composite scaffolds in inducing the osteogenic differentiation of MSC in the absence of external osteogenic stimuli.

The differentiation of MSC into osteoblasts is characterized by the coordinated expression of key genes during the various differentiation stages, from osteoprogenitor cells, through preosteoblasts, and finally into mature bone-building cells^65^. Evaluating the expression of these genes is fundamental for better understanding the impact of the 3D scaffolds on MSC osteogenic differentiation. In this study, the expression of BMP-2, runt related gene-2 (RUNX-2), osteocalcin (OCN), and type I collagen (COL1A1) was investigated since they are known to play a key role during osteoblast generation. Specifically, BMP-2 is a bone morphogenic protein belonging to the TGF-β family, a growth factor essential for guiding MSC differentiation towards osteoblasts, through the SMAD-signaling pathway^83^. RUNX-2 is a key transcriptional factor for initiating the osteogenic differentiation. Its expression is stimulated by BMP-2 and regulates the expression of major bone matrix genes (osteocalcin, osteopontin, bone sialoprotein, ALP and others). Usually, RUNX-2 is initially expressed in preosteoblasts, and it is further upregulated in immature osteoblasts. However, after the maturation of the cell, the RUNX-2 expression decreases^65,84^. COL1A1 gene is considered an early marker of osteoblast, which is upregulated during the transformation of osteoprogenitor into preosteoblasts^24,85^. Finally, OCN is expressed in mature osteoblasts during the late osteogenic differentiation stages. OCN is a calcium-binding protein intrinsic to the organic matrix of bones^86^.

**Figure 8:**
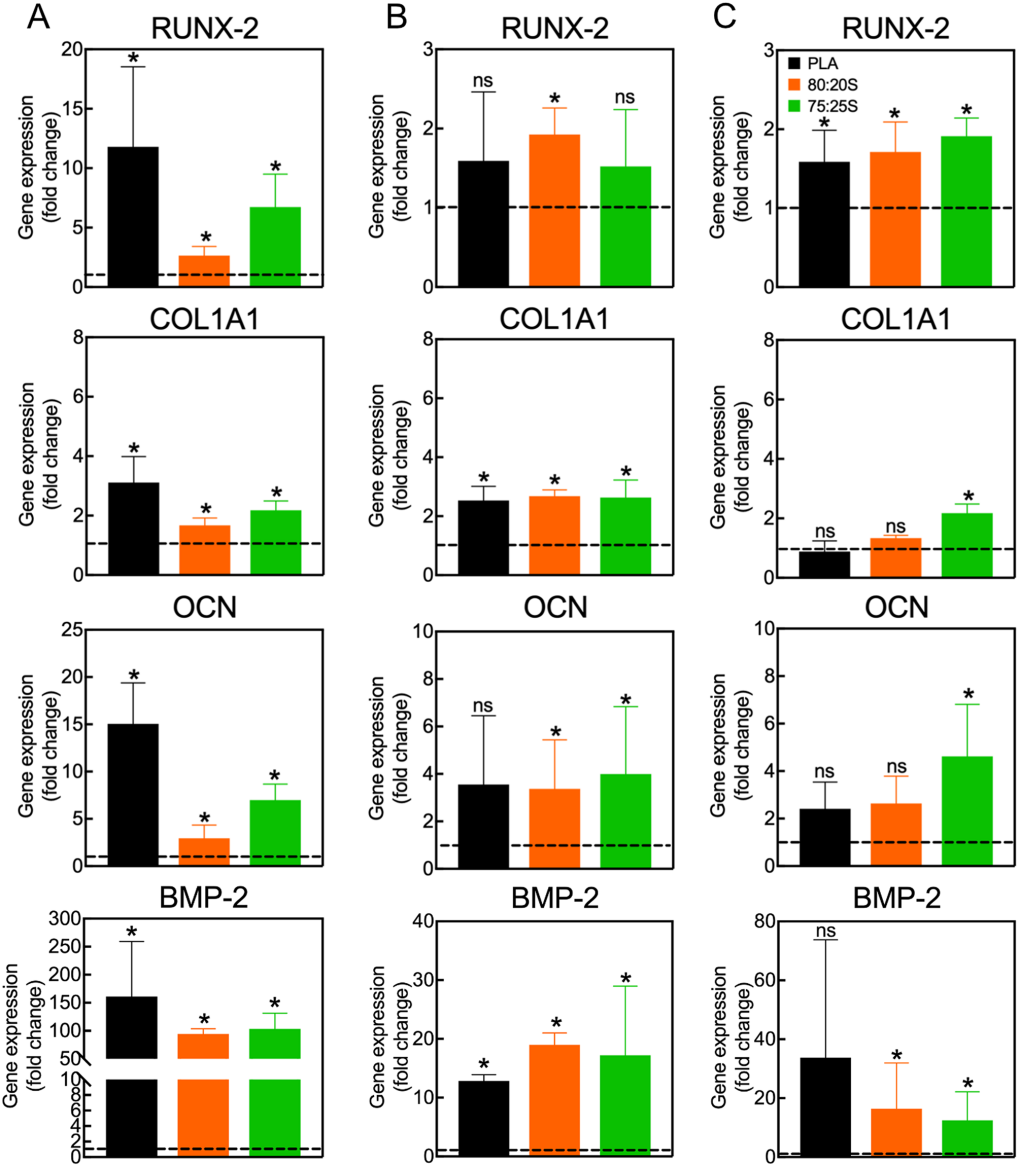
Quantification of gene expression in MSC cultured on 3D scaffolds under growth conditions for 7, 14 or 21 days (**A**–**C**, respectively). Data show the mean ± SD of two independent RT-PCR measurements. All values were normalized to GAPDH expression. Gene expression in control cells cultured on tissue culture dishes without scaffolds was set to one (dashed lines). *p < 0.05, *ns* non-significant. Please note that the statistical analysis refers to the comparison with the control.

MSC on 3D-printed PLA/HA scaffold in growth medium showed an increase of BMP-2, RUNX-2, OCN and COL1A1 expression compared to controls without scaffold (Fig. 8). The same kinetics of gene expression was observed when MSC were exposed to osteogenic stimuli (Fig. S12).

BMP-2 expression was prominently upregulated after 7 days, with a fold change of 100-times compared to control in growth medium. Over time, BMP-2 expression by 20-times, in accordance with the expected BMP-2 expression profile. Similar outcomes were observed for osteogenic medium. RUNX-2 had the peak of upregulation at day 7 of culture in growth medium, and then expression decreased after 14 and 21 days. However, for the osteogenic medium, the peak of RUNX-2 expression was found after 14 days of culture, followed by a decrease after 21 days. The results obtained in growth medium suggest that the 3D-printed scaffolds may lead to an anticipation of the immature differentiation stages, in the absence of other osteogenic factors, such as dexamethasone, ascorbic acid, and β-glycerophosphate. COL1A1 expression was essentially unchanged over time for both conditions, even though the COL1A1 expression was superior for 3D scaffolds than for the control in growth medium. Nevertheless, small changes in gene expression during osteogenesis tend to be sufficient to drive phenotypic changes on the cells^87^. Finally, the expression of OCN enhanced over time, achieving the peak of expression at day 21 for both media, revealing that, under both conditions, the cells were able to reach the mature osteoblast stage.

It is interesting to note that, when comparing the effect of the different 3D scaffolds on the expression of early-stage osteogenic genes, like RUNX-2, BMP-2 and COL1A1, or ALP activity, no significant difference in gene expression was found. These observations suggest that the chemical composition of the scaffolds is likely not the only feature that influences the initial stages of the osteogenic process. In this context, it is known that microstructural, mechanical, and morphological properties play fundamental roles at the beginning of osteogenesis^83,88,89^. Therefore, neat PLA scaffolds support the adhesion and proliferation of MSC and the initial formation of ECM, as indicated by the increase of ALP activity and high levels of RUNX-2, BMP-2 and COL1A1 expression. However, the expression of the late genes, like OCN, and ECM mineralization were not observed, suggesting that this kind of scaffolds could likely act as osteoconductive scaffolds, providing a suitable substrate for cellular activity^90^. On the other hand, the PLA/HA scaffolds not only supported adhesion, proliferation and initial formation of ECM, but also promoted the expression of the late gene (OCN) and ECM mineralization in growth medium after 21 days of culture. Collectively, our observations indicate that 3D-printed PLA/HA scaffolds efficiently support and promote the osteogenic differentiation of MSC also in the absence of any classical osteogenic stimuli, thereby showing an excellent potential as bone replacements. These results are in line with the observations that also PLGA-based scaffolds combined with ceramics, including HA, can support osteogenic differentiation of MSC in the absence of classic soluble osteogenic stimuli^29,30,32^.

## Conclusions

In this work, we developed bioactive 3D-printed PLA scaffolds containing high HA contents with potential application as bone tissue replacements. All 3D-printed composite scaffolds exhibited mechanical properties compatible with those of trabecular bone and additionally exhibited excellent biocompatibility towards DC and MSC. To the best of our knowledge, this was the first report on the interaction of DC with 3D-printed PLA/HA scaffolds and the immunological inertness of all composites toward these important immune cells. The PLA scaffolds with 20% HA showed adequate degradation rate and porosity and induced osteogenic differentiation, which allowed for the formation of mineralized ECM of cells, even in absence of osteogenic stimuli. Under osteogenic conditions, the PLA/HA scaffolds boosted the osteogenic response of MSC. Future studies should be aimed at better understanding how 3D-printed PLA/HA scaffolds can induce osteogenic differentiation of human MSC thus making them suitable as bone replacements for in vivo applications.

## Supplementary Information

Supplementary Information 1.

Supplementary Video 1.
