# Supplementary material for: PLA/Hydroxyapatite scaffolds exhibit in vitro immunological inertness and promote robust osteogenic differentiation of human mesenchymal stem cells without osteogenic stimuli
Source: Sci Rep. 2022 Feb 11;12:2333. doi: 10.1038/s41598-022-05207-w (PMC8837663; doi:10.1038/s41598-022-05207-w)
Supplement: Supplementary file 1 — Supplementary Information 1. [file 41598_2022_5207_MOESM1_ESM.docx]

*Supporting information for*: **Hydroxyapatite/PLA scaffolds exhibit *in vitro* immunological inertness and promote robust osteogenic differentiation of human mesenchymal stem cells without osteogenic stimuli**

Marcela P. Bernardo; Bruna C. R. da Silva; Ahmed E. I. Hamouda, Marcelo A. S. de Toledo; Carmen Schalla; Stephan Rütten; Roman Goetzke; Luiz H. C. Mattoso; Martin Zenke; Antonio Sechi

1. **MATERIALS**

Chloroform was purchased from Synth (Brazil). MTT (3-[4,5-dimethyl-2-thiazol]-2,5-diphenyl-2H-tetrazolium bromide) was purchased from Thermo Fisher, Germany. 4-nitrophenol (reference number 48549), phosphatase substrate (reference number 4744) and alkaline buffer solution (reference number 9226) were purchased from Sigma-Aldrich (St. Louis, MO, USA).

1. **METHODS**

*2.1 Characterizations*

The 3D printed composites were evaluated for their thermal properties by differential scanning calorimetry (DSC) carried out with Q100 Series instrument (TA instruments). Two heating/cooling scans were performed using hermetic aluminum pans under N_2_ atmosphere with flow of 60 mL.min^−1^ and heating rate of 10 °C min^−1^. The first scan was recorded from 25 °C to 200 °C (material's thermal history removal), followed by a cooling to -50 °C at 10 °C min^−1^. The second scan was performed from -50 °C to 200 °C. The crystallinity degree was calculated according to the following equation [B. Coppola, N. Cappetti, L. Maio, P. Scarfato, L. Incarnato. 3D printing of PLA/clay nanocomposites: Influence of printing temperature on printed samples properties, Materials. 10 (2018) 1-17. 10.3390/ma11101947]:

(1)

$$X=(({\Delta H}_{m}-\Delta H_{c})/H_{o}(1-W_{HA}))X 100\%$$

where, ΔH_m_ is the melting enthalpy of the sample, ΔH_c_ is the cold enthalpy of the sample, W_HA_ is the weight fraction of HA, H_o_ is the melting enthalpy of the fully crystallized PLA, equal to 93.7 J/g [M. Persson, G.S. Lorite, S.W. Cho, J. Tuukkanen, M. Skrifvars, Melt spinning of poly(lactic acid) and hydroxyapatite composite fibers: Influence of the filler content on the fiber properties, ACS Appl. Mater. Interfaces. 5 (2013) 6864–6872. https://doi.org/10.1021/am401895f] and W_HA_ is the HA content.

Thermogravimetric (TGA) analysis was performed with a TA (Q500 Series) equipment. The samples were dried for 24 h before the TGA experiments. The experiments were conducted using an aluminum pan in the temperature range from 25 °C to 500 °C (heating rate of 10 °C min^−1^) using N_2_ atmosphere (60 mL min^−1^). The porosity content of the printed scaffolds was evaluated by microtomography (µCT-scan) on a SkyScan model 1172 with filter of aluminum. The projections were acquired with a total rotation of 180° in steps of 0.2° and spatial resolution of 4.95 µm. Subsequently, the projections were reconstructed as bidimensional images with the CTan software (SkyScan) with smoothing 5, ring artifact correction of 10 and beam hardening correction of 60%. The hydrophilic/hydrophobic surface characteristics of the scaffolds were investigated by contact angle measurements using a contact angle meter (KSV Instruments – Helsinki, Finland). Images of a water drop with an estimated volume of 5 µL were acquired and analyzed by a CCD video camera. The initial (t = 0 s) and equilibrium (t = 25 s) contact angles were calculated by averaging 5 individual measures (n = 5) from 5 different 3D printed scaffold specimens.

*2.2 Tests of Degradation*

A 0.9% NaCl solution with pH adjusted to 7.4 (with 0.1 M NaOH and 0.1 HCl) was used to simulate the body fluid. 3D-printed discs (10 mm x 1 mm, diameter x height) of each polymer composite evaluated were precisely weighted before immersed in inert plastic tubes containing 13 ml of the saline solution. The tubes were maintained at 37 °C under 100 rpm agitation. The study was performed for 11 weeks. The experiments were performed individually and in triplicate. Every week, tubes were withdrawn off the experiment. The discs were washed with distillated water and dried under vacuum for 24 h at 37 °C. Afterwards, the discs were weighted and the weight loss rate (WLR) was calculated as follows:

(2)

$$WLR= ((Wo-W)/Wo) X 100\%$$

where, W_o_ and W are the initial weight and weight at the degradation time point, respectively.

Every week, the pH, phosphate and calcium contents of the saline solution were also analyzed. The phosphorus concentration was determined according to a previously reported procedure [L. Drummond, W. Maher, Determination of phosphorus in aqueous solution via formation of the phosphoantimonylmolybdenum blue complex Re-examination of optimum conditions for the analysis of phosphate, Anal. Chim. Acta. 302 (1995) 69–74.]. The calcium concentration was determinate by atomic absorption on a PinAAcle 900T (Perkin Elmer), using flame as atomization mode (Nitrous oxide 6 L min^-1^; Acetylene 7.5 L min^-1^), wavelength of 422.67 nm and slit width of 0.7 nm.

*2.3 Generation and culture of dendritic cells*

Briefly, bone marrow progenitor cells were cultured at a density of 2 x 10^6^ cells/mL in RPMI 1640 medium supplemented with 10% FCS, 2 mM L-glutamine, 100 µg/mL streptomycin, 100 U/mL penicillin (all from Invitrogen Life Technologies, Germany), 50 mM 2-mercaptoethanol, recombinant murine SCF (100 ng/mL), 25 ng/mL Flt3 ligand (PeproTech, Germany), 40 ng/mL recombinant long-range IGF-1 (Sigma-Aldrich, Germany), 5 ng/mL rIL-6/ soluble IL-6R fusion protein (hyper-IL-6; kindly provided by S. Rose-John), 20 U/mL recombinant mouse GM-CSF, and 1 mM dexamethasone. After 7 days of culture, BMPC were differentiated into DC using the above medium supplemented only with 250 U/mL recombinant murine GM-CSF. Dendritic cells were activated with 1 µg/mL lipopolysaccharides (LPS) from *Escherichia coli* 0127:B8 (Sigma-Aldrich, Germany) for 24 or 48 h.

*2.4 Culture of* *human mesenchymal stem cells (MSC)*

Briefly, cells were flushed from the bone and cultured in parallel in proliferation medium consisting of Dulbecco’s modified Eagle’s medium (1 g/L glucose; Sigma-Aldrich, St. Louis, MO), 1% penicillin/streptomycin and 1% L-glutamine (all from Invitrogen Life Technologies, Germany), and supplemented with 10% human platelet lysate (hPL). hPL pools consisted of at least five lysates to reduce variation, and coagulation was prevented by 0.61 IU unfractionated heparin (Ratiopharm, Ulm, Germany). For the osteogenic conditions, osteogenic differentiation medium was included, which was composed of DMEM (1 g/L glucose) supplemented with 200µM ascorbic acid-2-PO_4_, 100 nM dexamethasone, and 10 mM β-glycerophosphate and 10% FCS (Lonza, Germany).

*2.5 In vitro biocompatibility*

Immature dendritic cells (3 × 10^5^ cells) were incubated on the scaffolds at 48-well plates. After 24h or 48h of incubation at 37 °C in a humidified 5% CO_2_ atmosphere, 10 μL MTT (5 mg/mL, PBS solution) solution were added to each well, and the cells were incubated for another 4 h. Afterwards, 100 μL of lysis solution (0.1 N of HCl in isopropanol) were added to each well with incubation at room temperature for 10 min. For the MSC, the cytotoxicity assay was conducted according to the ISO 10993-5:2009 protocol. MSC were cultivated in the proliferation medium at 37 °C in a humidified 5% CO_2_ atmosphere incubator. To obtain the sample extracts, the scaffolds were incubated for 1, 2, and 3 weeks in the proliferation medium. The cells were seeded at a density of 2 × 10^4^ cells per well and stored overnight under proper conditions. The cell medium was replaced with the scaffold extract, and the cells were incubated in the humidified incubator for 24 h before the MTT assay. Next, 10 μL MTT solution (5 mg/mL, PBS solution) were added to each well and incubate for 4h. Afterward, the MSC were lysed following the same procedure for DC. Cells seeded on empty wells and cultivated with the proliferation medium was used as a control. For both cells, the absorbance was recorded at 570 nm for each well with a microplate reader (Molecular Devices SpectraMax M2e). Viability of cells in contact with the scaffolds (or extracts) was expressed related to untreated cells (set to 1). All the experiments were repeated in triplicate using five-well replicates for each repetition.

*2.6 MSC differentiation on scaffolds*

For ALP (alkaline phosphatase) quantifications the absorbance was measured at 405 nm using a microplate reader (Molecular Devices SpectraMax M2e). The results were normalized to the total protein content and shown as fold expression of untreated control cells (set to 1). All the experiments were repeated in triplicates.

**FIGURES**


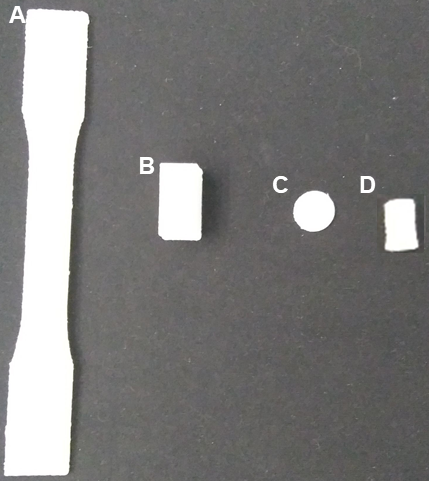


**Fig. S1.** 3D-printed scaffolds used for A) tensile mechanical tests; B) compression mechanical tests; C) *in vitro* degradation and D) porosity experiments.


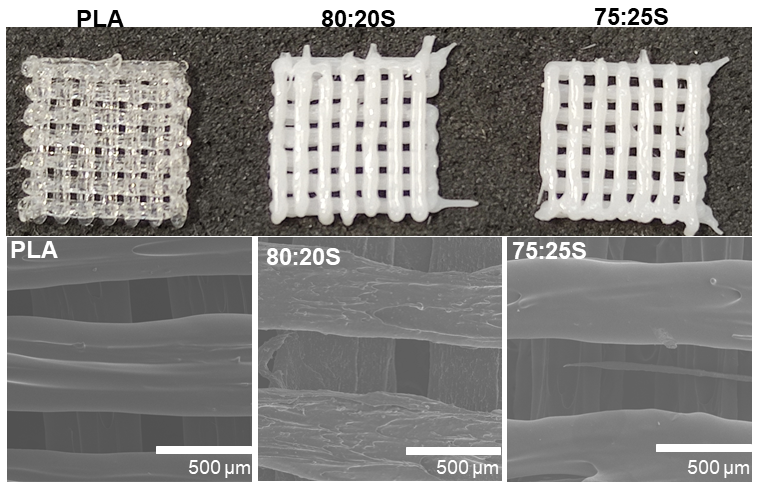


**Fig. S2**. 3D-printed scaffolds (7 mm x 7 mm x 1 mm, height x length x thickness, with pore size of 300µm) used for all biological experiments.


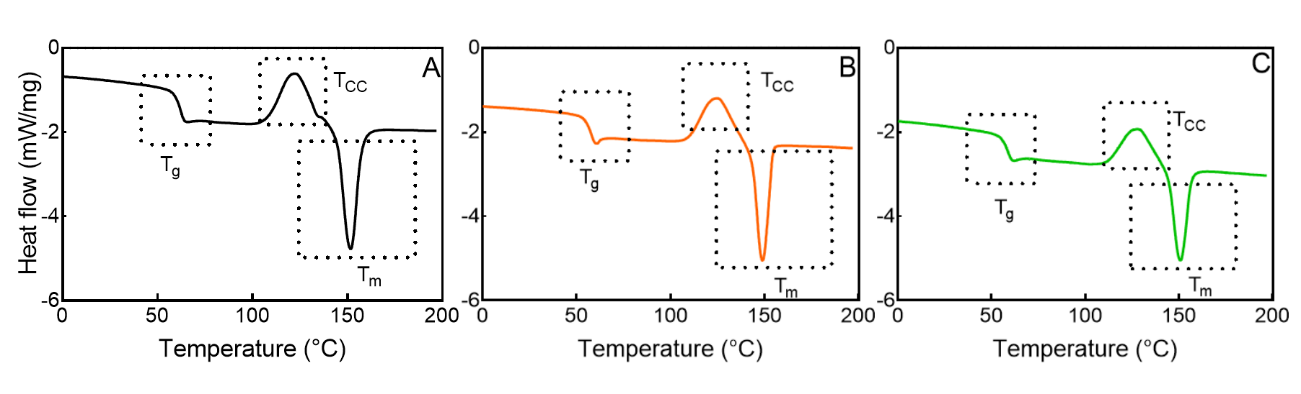


**Fig S3.** DSC curves for the second heating for A) PLA; B) 80:20 S and C) 75:25 S.

T_g_ = glass transition temperature, T_cc_ = cold crystallization temperature, T_m_ = melting temperature

**Fig. S4.** Thermogravimetric analysis curves of scaffolds A) Neat PLA and polymer composites B) 80:20 S; C) 75:25 S.


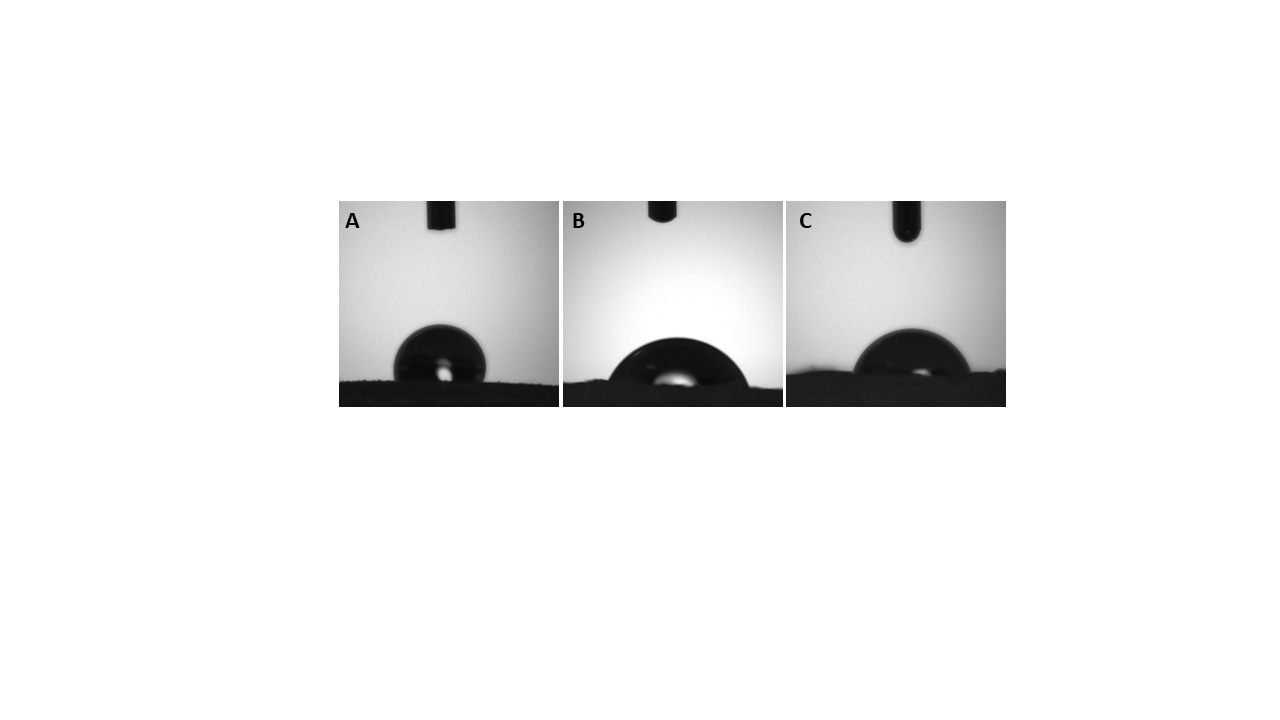


**Fig S5**. Images of water contact angle at the 3D-printed scaffolds surface at 25s for A) PLA; B) 80:20 S; C) 75:25 S


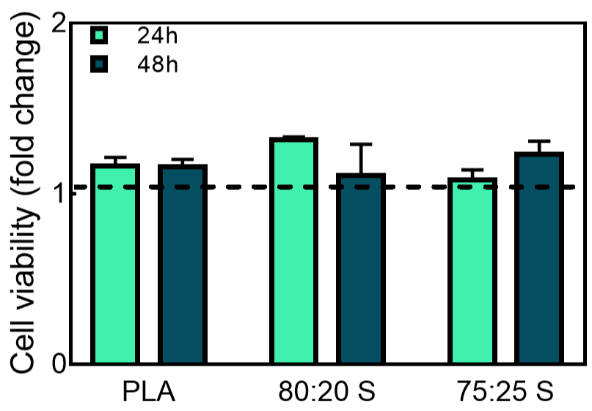


**Fig S6.** Biocompatibility of 3D-printed scaffolds to dendritic cells after 24 and 48 h of contact. Cells cultivated without materials were used as control (cell viability set at 1).

**Fig S7.** Biocompatibility of 3D-printed scaffolds towards MSC after 7, 14, and 21 days. Cells cultivated without materials were used as a control (cell viability set at 1).


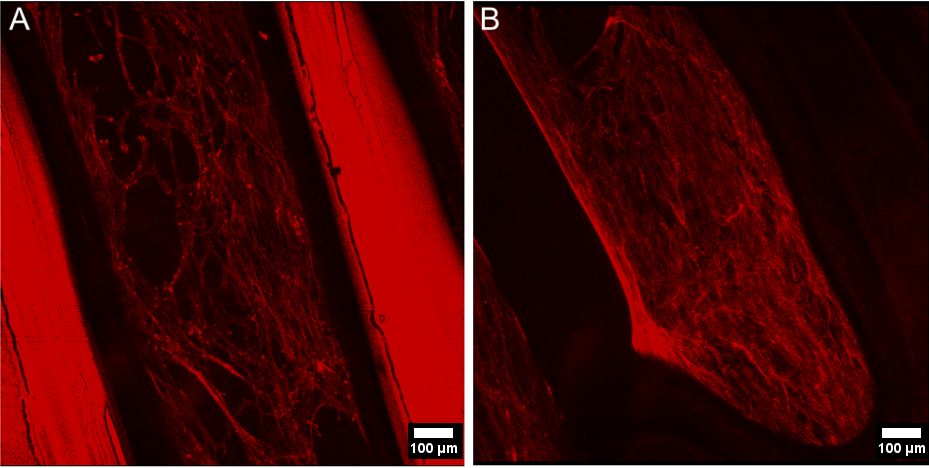


**Fig. S8.** Representative confocal microscopy images of MSC growing on PLA 3D-printed scaffold surface after 14 (A) and 21 (B) days of cultivation. Cells were visualized with Alexa 647-conjugated phalloidin.


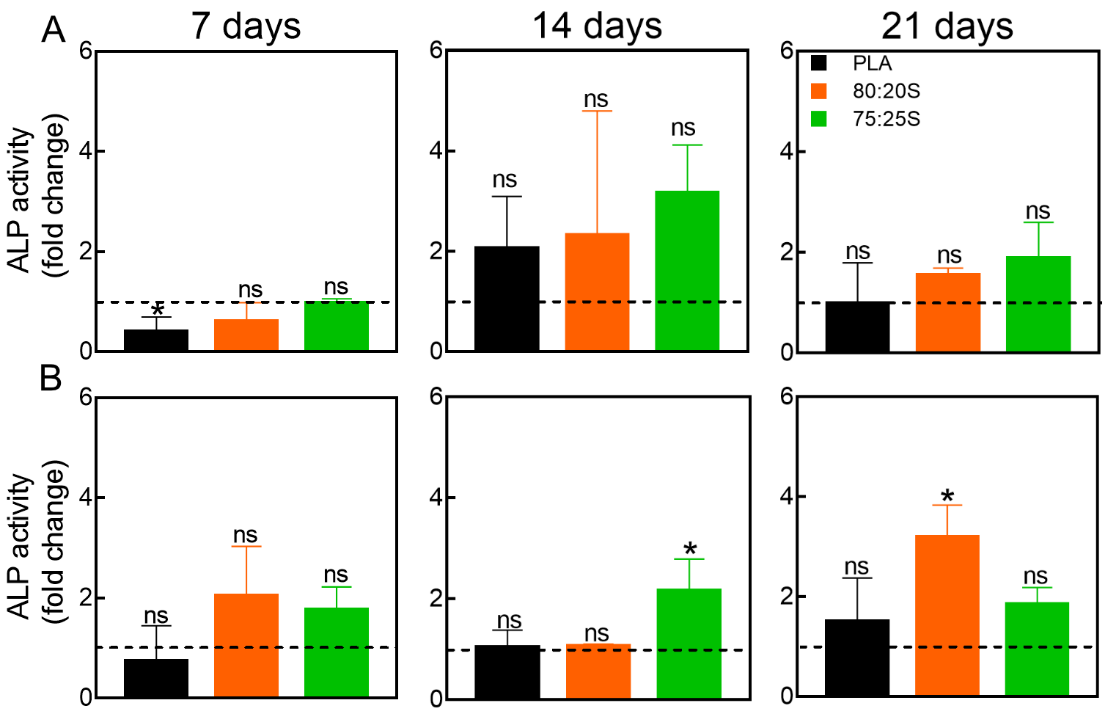


**Fig. S9.** Alkaline phosphatase activity of MSC cultured for 7, 14 and 21 days under proliferation or osteogenic conditions. Dashed lines (set at 1) represent the control for each condition. ns: no statistical difference (**p* < 0.05). Please note that the statistical analysis refers to the comparison with the control.


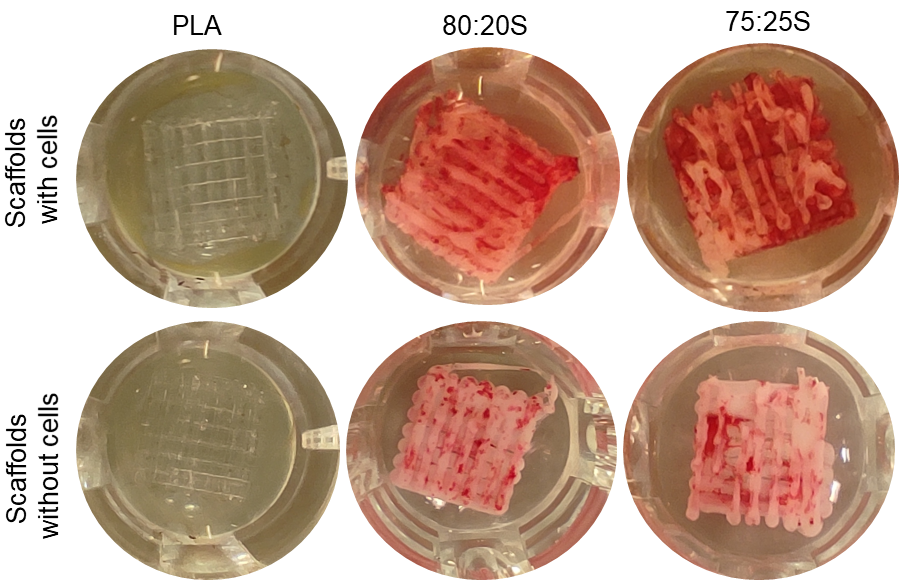


**Fig. S10.** Representative images of MSC seeded on 3D-printed scaffolds for 21 days under growth conditions and then stained with alizarin red (upper panels). Lower panels show alizarin red stained 3D scaffolds devoid of MSC.


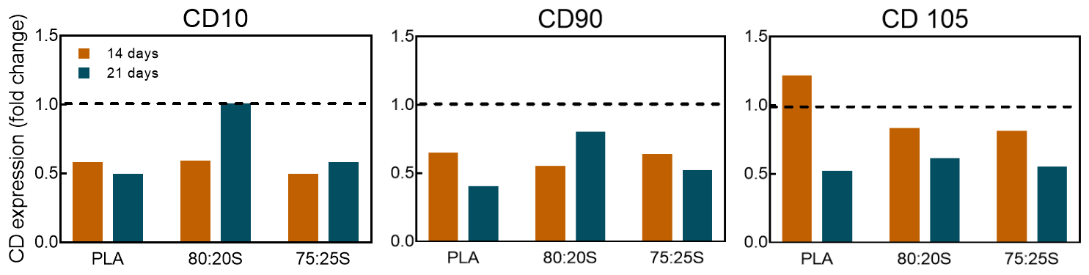


**Fig. S11.** Flow cytometry analysis of the expression of CD10, CD105 and CD90 in MSC cultivated on 3D-printed scaffold for 14 and 21 days under osteogenic conditions. Dashed lines represent the expression of the surface markers in control, untreated cells (set to 1).


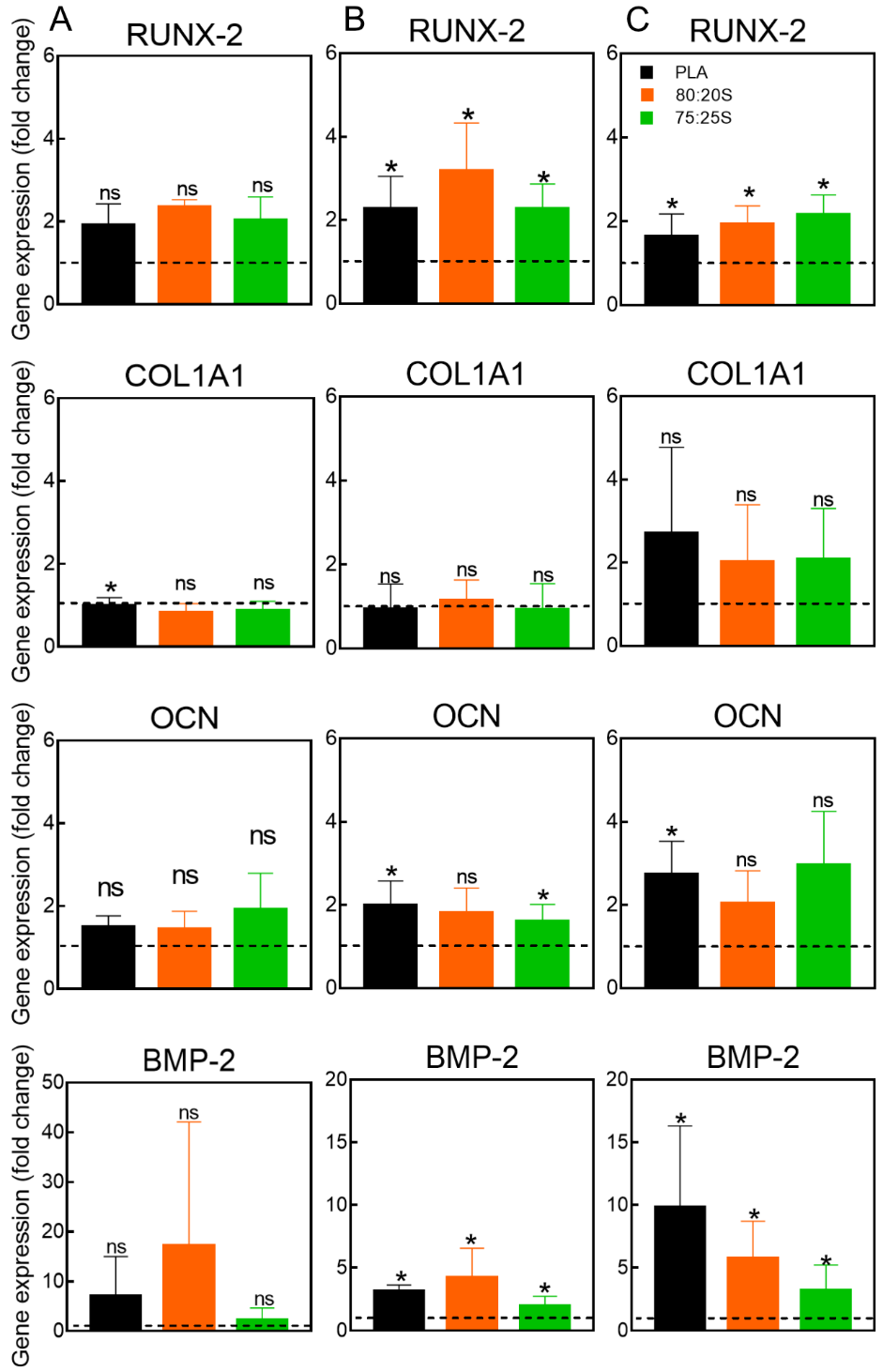


**Fig S12.** Quantification of gene expression in MSC cultured on 3D scaffolds under osteogenic conditions. Data show the mean±SD of two independent RT-PCR measurements. All values were normalized to the expression levels of GAPDH. Gene expression in control cells (cultured on tissue culture dishes) was set to one (dashed lines). **p*<0.05, ns: non-significant. Please note that the statistical analysis refers to the comparison with the control.

**TABLES**

**Table S1.** PLA:HA contents of composite filaments and sample codes.

| **PLA:HA proportion (Mass, %)** | **Label samples** |
| --- | --- |
| 100:0 | PLA |
| 80:20 | 80:20 S |
| 75:25 | 75:25S |

**Table S2.** Sequences of the primers used for RT-PCR analyses.

| Target | Primers |
| --- | --- |
| GAPDH | Fwd:5’-ACCTGCCAAGTATGATGACATCA-3’  Rev:5’-GGTCCTCAGTGTAGCCCAAGAT-3’ |
| IL-1β | Fwd:5’-CCAAAAGATGAAGGGCTGCT-3’  Rev:5’-TCATCAGGACAGCCCAGGTC-3’ |
| IL-6 | Fwd:5’-TCTGGGAAATCGTGGAAATGAG-3’  Rev:5’-ATTGGATGGTCTTGGTCCTT-3’ |
| IL-10 | Fwd:5’-TTTGAATTCCCTGGGTGAGAA-3’  Rev:5’-ACAGGGGAGAAATCGATGACA-3’ |
| IL-12p40 | Fwd:5’-TTGCTGGTGTCTCCACTCAT-3’  Rev:5’-GGGAGTCCAGTCCACCTCTA-3’ |
| RANTES | Fwd:5’-TGCTCCAATCTTGCAGTCGT-3’  Rev:5’-ACACACTTGGCGGTTCCTTC-3’ |
| RUNX-2 | \| Fwd: 5’-ACCCAGAAGGCACAGACAGAAG-3’ \| \| --- \| \| Rer: 5’-AGGAATGCGCCCTAAATCACT-3’ \| |
| Col1A1 | \| Fwd: 5’-ACATGGACCAGCAGACTGGCA-3’ \| \| --- \| \| Rer: 5’-TCACTGTCTTGCCCCAGGCT-3’ \| |
| BMP-2 | \| Fwd: 5’-CAACACTGTGCGCAGCTTCCACC-3’ \| \| --- \| \| Rer: 5’-GTGGGCCACTTCCACCACGAATC-3’ \| |
| OCN | \| Fwd: 5’-AGGGCAGCGAGGTAGTGA-3’ \| \| --- \| \| Rer: 5’-CCTGAAAGCCGATGTGGT-3’ \| |

**Table S3.** Porosity of 3D-printed PLA/HA scaffolds ^α^

| **Samples** | **Porosity (%)** |
| --- | --- |
| PLA | 5.7 (0.5)^b^ |
| 80:20 S | 17.6 (0.5)^a^ |
| 75:25 S | 16.6 (0.8)^a^ |

^*α^ Mean value (standard error). Means in the same column bearing the same letter are not significantly different (p > 0.05).

**VIDEO**

**V1:** 3D rendering of MSCs plated on PLA scaffold for 7 days stained with Alexa 647-conjugated phalloidin.
